# Patients’ Perception of Telemedicine in a Large Urban Inner-City Emergency Department: A Cross-Sectional Survey

**DOI:** 10.7759/cureus.11091

**Published:** 2020-10-22

**Authors:** Eric Sechrist, Fanglong Dong, Carol Lee, Kaitlin Chon, Arianna Neeki, Lori Winston, Rodney Borger, Michael M Neeki

**Affiliations:** 1 Emergency Medicine, Arrowhead Regional Medical Center, Colton, USA; 2 Emergency Medicine, Kaweah Delta Medical Center, Visalia, USA

**Keywords:** telemedicine, emergency department, low socioeconomic

## Abstract

Introduction

Telemedicine has the potential to ease emergency department (ED) overcrowding, improve ED throughput, and decrease the cost of medical care. Much of the current knowledge of telemedicine systems focuses on bringing more specialty care to the ED or improving access in rural areas. Limited research exists on patients’ perception of telemedicine in an urban ED.

Methods

A survey exploring perceptions of telemedicine encounters was distributed to both providers and patients following mirrored encounters between October 2015 and August 2016. Chi-square analysis was conducted to identify associations between factors and openness to telemedicine from the patients’ perspective.

Results

A total of 174 patients were included in the analysis. Factors associated with patient willingness to try telemedicine included: having access to a tablet with internet (p=0.0023), having access to a tablet with camera (p=0.0025), having downloaded apps in the past (p=0.0028), having used an app in the past (p<0.0001), and had frequent video chat in the past (p=0.0142).

Conclusion

With widespread access to smartphones with internet connectivity and pressing demands for healthcare services, telemedicine may provide a potential solution to low acuity medical care needs.

## Introduction

In the United States, between 2006 and 2011, the total amount of annual visits to emergency departments (EDs) increased by 4.5% from 40,200 to 42,100 per 100,000 population, which may include repeated visits by the same individual [1]. In a 2011 analysis of ED visits resulting in discharge, the most common conditions included fever in infants, otitis media, viral illnesses, superficial injuries, abdominal pain, back pain, headache, and urinary tract infections [2]. Many of these low acuity conditions could potentially be diagnosed and treated using telemedicine [2-4].

Telemedicine, the provision of health care via telecommunication and information technology, is a significant and rapidly growing component of healthcare in the United States [5]. The swift evolution of information technology and widespread use of the internet since the 1980s has pushed telemedicine from a 40-year-old concept into present-day application [6,7]. This change has been facilitated by high-speed telecommunication which allows for real-time transfer of information, images, and videos [6]. Many medical specialties, including EDs, have considered how the application of telemedicine may become an important alternative to in-person healthcare treatment [6]. Additionally, telemedicine may be able to eliminate the distance and cost barriers that hinder medical aid today, therefore leading to improved access to medical services [6,8]. Furthermore, telemedicine has been suggested as a useful tool to offset physician shortages [9,10].

Telemedicine may have the potential to safely ease ED overcrowding, reduce ED ambulance transports, improve ED throughput by integrating specialty services, and decrease the cost of medical care [8,11-16]. Rademacher and colleagues reported that using telemedicine for screening patients in the ED significantly decreased the number of patients left without being seen [17]. In many urban areas, the ED has become the primary facility for both insured and uninsured patients to obtain healthcare due to accessibility outside of regular business hours. In addition, patients living in the poorest communities had 25% higher average ED revisit rates compared to patients residing in the wealthiest areas [18]. Telemedicine in the ED setting may allow working-age patients to access care in without missing work and may improve disparities in care [17,19].

Much of the current knowledge of telemedicine systems focuses on bringing more specialty care to the ED or the use of telemedicine in small rural hospitals [6,13,20-22]. Limited research exists on patients' perceptions, economic effects, and process outcome of patients using telemedicine for low acuity care in lieu of an ED visit [7,23]. A systematic review of studies conducted on telemedicine use in EDs discovered that the majority of studies examined the application of telemedicine in regard to provider-to-provider communication and only two studies explored patient-to-provider communication [7]. Additionally, low acuity care studies revealed a lack of evidence in regard to user perceptions when using telemedicine [7].

The current study aims to assess patients’ perceptions of a telemedicine system in a large, urban inner-city ED with a primarily low socioeconomic status. Furthermore, this study intends to identify some perceived barriers to the effective implementation of telemedicine in an urban ED.

## Materials and methods

A cross-sectional, prospective study was conducted via questionnaires targeting both providers’ and patients’ perceptions of telemedicine between October 2015 to August 2016 at Arrowhead Regional Medical Center (ARMC) in San Bernardino County, California. Questionnaires were distributed to both providers and patients for mirrored encounters via SurveyMonkey. The ARMC ED is one of the busiest EDs in the state of California with more than 100,000 annual visits [24]. San Bernardino County is the largest county in the United States with an estimated population of 2 million with approximately 49% of Hispanic or Latino ethnicity [25].

Data were collected through a questionnaire on SurveyMonkey (Appendix A). The survey consisted of 20 questions, eight of which were answered by the ED provider (attending physician, resident physician, or physician assistant) and the remainder of which were answered by participating patients. The questionnaires addressed patient demographics, access to technology, and computer literacy (Appendix A). The survey also addressed both provider and patient perceptions regarding telemedicine versus in-person healthcare for the visit. Research assistants interacted with ED providers and patients with low acuity illness, and in real-time entered participants' responses into SurveyMonkey for data collection. Patients were blinded to providers’ answers and vice versa. Additionally, if a patient had difficulty with literacy, the research assistants read the questions to the patients. The questionnaires were available in English and Spanish and a certified translator was provided when necessary. The questionnaires were completed at various times throughout the day, covering all operational hours of the ED. This study was reviewed and approved by the Institutional Review Board at ARMC.

All statistical analyses were conducted using the SAS software for Windows, version 9.3 (SAS Institute Inc., Cary, NC). Descriptive statistics were presented as frequencies and proportions for categorical variables. Chi-square analysis was conducted to assess the association between various variables (including demographic variables and access to technology) and willingness to try telemedicine. All statistical analyses were two-sided. P-value <0.05 was considered statistically significant.

## Results

Among the initial 188 participants who were surveyed, 14 participants were excluded (seven for incomplete surveys and an additional seven due to admission to hospital). A final 174 (92.6%) patients were included in the analysis. Table 1 presented the demographic summary of the patients. More than half (69.5%) of the patients were willing to try telemedicine for the visit. Half of the patients were female (54%), Latino or Hispanic (59.2%), between age 17 and 39 (57.5%), ≤ high school education (64.9%), were employed (47.1%), had state Medicaid health insurance (80.9%), and spoke English (67.2%) (Table 1).

**Table 1:** Crosstab analysis of demographic variables associated with openness to telemedicine in general* *All values in Table 1 are presented as N (%). Percentage was calculated as column percentage.

	Overall (N=174)	Patients expressed unwillingness to participate in Telemedicine (n=53)	Patients expressed willingness to participate in Telemedicine (n=121)	P-value
Male Percentage	80 (46%)	28 (52.8%)	52 (43%)	0.2300
Age Group				0.2851
17 to 39	100 (57.5%)	27 (50.9%)	73 (60.3%)	
40-59	59 (33.9%)	19 (35.9%)	40 (33.1%)	
60 or older	15 (8.6%)	7 (13.2%)	8 (6.6%)	
Education				0.1404
<= High School	113 (64.9%)	40 (75.5%)	73 (60.3%)	
Some College	58 (33.3%)	12 (22.6%)	46 (38%)	
Graduate	3 (1.7%)	1 (1.9%)	2 (1.7%)	
Race				0.5384
White	25 (14.4%)	5 (9.4%)	20 (16.5%)	
Black or African-American	27 (15.5%)	7 (13.2%)	20 (16.5%)	
Latino or Hispanic	103 (59.2%)	35 (66%)	68 (56.2%)	
Other	19 (10.9%)	6 (11.3%)	13 (10.7%)	
Employment				0.1381
Employed	82 (47.1%)	21 (39.6%)	61 (50.4%)	
Not employed	69 (39.7%)	22 (41.5%)	47 (38.8%)	
Disabled	15 (8.6%)	4 (7.6%)	11 (9.1%)	
Retired	8 (4.6%)	6 (11.3%)	2 (1.7%)	
Health insurance				0.2281
State Medicaid	135 (80.8%)	42 (80.8%)	93 (80.9%)	
Private Insurance	6 (3.6%)	2 (3.9%)	4 (3.5%)	
Medicare	4 (2.4%)	3 (5.8%)	1 (0.9%)	
No health insurance	22 (13.2%)	5 (9.6%)	17 (14.8%)	
Language				0.0841
English	117 (67.2%)	30 (56.6%)	87 (71.9%)	
Spanish	49 (28.2%)	21 (39.6%)	28 (23.1%)	
Other	8 (4.6%)	2 (3.8%)	6 (5%)	

Regarding patient’s access to technology, three areas were assessed: accessibility to a cell phone, tablet, and computer (Table 2). A majority (83.3%) of patients had access to a cell phone with internet access, and 93.1% had access to a cell phone with camera. Secondly, 56.9% of patients had access to a tablet with internet, and 54.9% of patients had access to a tablet with camera. Lastly, 58.1% of patients had access to a computer with internet, and 43.4% had access to a computer with camera. Furthermore, 82.8% had downloaded an app in the past, 82.1% had used an app in the past, and 62.6% had experience with video chatting in the past. Factors associated with patients’ willingness to try telemedicine in general included: having access to a tablet with internet (p=0.0023), having access to a tablet with camera (p=0.0025), having downloaded apps in the past (p=0.0028), having used an app in the past (p<0.0001), and having had frequent video chats in the past (p=0.0142). Preliminary evidence indicates that demographic variables, such as age, income level, education level, and so on, were generally not strongly associated with willingness to try telemedicine.

**Table 2:** Crosstab analysis of technology-related variables associated with openness to Telemedicine in general* *All values in Table 2 are presented as N (%). Percentage was calculated as column percentage; **one response was missing for this question.

	Overall (N=174)	Patients expressed unwillingness to participate in Telemedicine (n=53)	Patients expressed willingness to participate in Telemedicine (n=121)	P-value
Have access to cellphone with internet	145 (83.3%)	40 (75.5%)	105 (86.8%)	0.0655
Have access to cellphone with camera	162 (93.1%)	49 (92.5%)	113 (93.4%)	0.8226
Have access to tablet with internet	99 (56.9%)	21 (39.6%)	78 (64.5%)	0.0023
Have access to tablet with camera	95 (54.9%)	20 (37.7%)	75 (62.5%)	0.0025
Have access to computer with internet	101 (58.1%)	25 (47.2%)	76 (62.8%)	0.0543
Have access to computer with camera	77 (44.3%)	23 (43.4%)	54 (44.6%)	0.8803
Downloaded app in the past	144 (82.8%)	37 (69.8%)	107 (88.4%)	0.0028
Used app in the past**	142 (82.1%)	34 (64.2%)	108 (90%)	<0.0001
Used video chat in the past	109 (62.6%)	26 (49.1%)	83 (68.6%)	0.0142

## Discussion

This study examined whether the patients’ perception of telemedicine would be feasible for patients in a large, urban ED with a primarily lower socioeconomic population. ARMC serves San Bernardino County, where nearly 18% of the population lives below the poverty level [26]. Utilizing health insurance as an indicator of socioeconomic status, this study showed that 80.9% of the patients were under state Medicaid coverage, which suggests that the majority of the participants qualify for low-income programs. With 69.5% of the participants being open to telemedicine, this study indicates that telemedicine may be an acceptable alternative to an ED visit for patients from a lower socioeconomic population.

Additionally, the current study showed the majority of surveyed patients have access to cell phone technology, and close to half have access to tablet and computer technology. Despite a regional poverty level of 17.6%, more than 80% of the patients in this study had cell phones with internet capability and a functional camera. Nationally, both cell phone and smartphone usage has steadily increased with 95% of American adults owning a mobile device and 77% of American adults owning a smartphone as of November 2016 [27]. For the Latino population, who present as the majority of the participants in this study and the surrounding region, the ownership and use of mobile technology generally reflect a similar upwards trend [26]. While there may be a variation in each individual’s proficiency in using technology, the wide ownership of mobile technology may have led to participants’ readiness to utilize telemedicine [28].

In order to correlate previous use of mobile technology with proficiency in using technology and willingness to utilize telemedicine, this study examined the past usage of mobile technology of the participants. Approximately 80% of the participants stated they had downloaded and used an app in the past, and approximately 60% had experience using video chat software. While the study did not stratify app use with the age of participants, it suggests that previous app and video chat use may be correlated with increased acceptance of telemedicine.

Reasons for the relatively high willingness to utilize telemedicine may be related to increased access for those in the low socioeconomic population. Previous studies have shown that access to telemedicine services generally results in increased equity among different socioeconomic groups in the pediatric population [17]. Causes for this change in low socioeconomic groups may include reduced missed work, easier and more convenient access to health care, and availability of health care access outside of traditional business hours [17,19].

While our study suggests the potential for telemedicine to improve healthcare access and alleviate utilization of the ED for low-acuity care, there may be potential limitations. Patient barriers may include financial concerns and health and technology literacy issues, both of which may hinder the sustainability of telemedicine. While reimbursable telemedicine visits may be favored, commercial telemedicine may incur additional health costs that could not be supported by a low-income population. Additionally, patients may lack familiarity with technology or have low health literacy, which may further complicate the use of telemedicine for conducting a remote visit. Proper reimbursement via state Medicaid programs and patient education may allow patients to overcome such barriers. Furthermore, judging from the majority of patients’ willingness to try telemedicine in this study, many tangible and intangible benefits such as scheduling convenience, less interference with work schedule and childcare, and eliminating transportation barriers may make this option more attractive to many of these patients [19].

Limitations

The generalizability of findings in the current study was limited by the sample size of 174 patients in the study within an 11-month period. The proportion of Hispanic (59.2%) in the current study was similar to the national statistics of the Hispanic population in San Bernardino County (61.9%) [25]. While our findings may be generalizable to urban ED demographics, they may have limited generalizability for higher socioeconomic demographics and those with private insurance. Future research is needed to explore the effects of income and insurance coverage in relation to user preferences of telemedicine. 

## Conclusions

With the onset of ubiquitous smartphone access, rapid and widely accessible internet connectivity, and pressing demands for healthcare services, telemedicine may provide a potential solution to the low acuity medical care needs of many communities, particularly those low socioeconomic communities. 

## Appendices

Appendix A - Questionnaire

1.   Is the patient admitted or discharged from the ED?

o   Discharged home

o   Discharge to facility (nursing home, long term care, jail)

o   Admitted to hospital

2.  How did the patient come to the ED?

o   Car, bus, or walking

o   Ambulance, helicopter, police

3.  Is the patient over 19, or is their caretaker available to consent to the survey and answer the questions?

o   Patients is over 18 and can answer the questions

o   Patients is under 19, but has caretaker available to consent and answer questions

o   Patients is demented, but has caretaker available to consent and answer questions

o   Patients is under 18 and a caretaker is not available

o   Patients is demented and a caretaker is not available

4. What kinds of health insurance does the patient have?

o   I do not have health insurance

o   Medical

o   Managed medical

o   Emergency medical

o   Kaiser

o   PPO

o   HMO

o   Anthem BlueCross

o   Healthnet

o   Other(please specify)_______________________________

5. Please type the name of the patient’s health insurance below

_________________________________________

6. As the treating ED provider, would you have been comfortable caring for this patient through a telehealth visit without the ability to physically touch/examine the patient?

o   I could have provided care with phone only interaction

o   I could have provided care with video conference interaction

o   I need physical presence and exam to provide care

7. Do you think the patient would have felt a telehealth interaction was adequate to meet their needs so they would not seek further care in the ED, urgent care, or with their primary care doctor in the next 48 hours?

o   I could have provided care with phone only interaction

o   I could have provided care with video conference interaction

o   I need physical presence and exam to provide care

8. Did the patient agree to participate in the survey?

o   Yes

o   No

9. Access to personally technology

No/Yes/Sometimes/Don't know

Do you have access to a cellphone?

Do you have access to a cellphone with internet connection?

Does your cell phone have a camera?

Do you have access to a tablet (iPad, Kindle Fire, Galaxy, etc) with internet connection?

Does your tablet have a camera?

Do you have access to a computer with internet connection?

Does your computer have a camera?

10. How often have you …

Never/Once/Several times/Frequently/N/A

Downloaded an app (like Facebook, games) onto your phone, tablet, or computer

Used an app on your phone, tablet or computer

Done a video chat (like Skype)

Been treated by a doctor on the phone

Been treated by a doctor online (phone, tablet, or computer)

11. If you were able to get immediate to a doctor over the phone or through video chat, where they could talk to you, understand what was going on, and prescribe appropriate medications and referrals would you have preferred to use that service of coming to the ED?

o   I would have preferred a video chat interaction rather than coming to the ED today

o   I do not believe a doctor could have met my needs over the phone or through video chat

o   Other (please specify)_____________________________________

12. If you were able to get immediate access to a doctor over the phone, how often would you need to use that service rather than seeing your regular doctor, urgent care, or the ED?

o   Never

o   Once a year

o   Twice a year

o   Once a month

o   Twice a month

o   Once a week

o   Twice a week 

13. Are you male or female?

o   Female

o   Male 

14. What is your age?

o   17 or younger

o   18-20

o   21-29

o   30-39

o   40-49

o   50-59

o   60 or older

15. What is the highest level of school you have completed or the highest degree you have received? 

o   Less than high school degree

o   High school degree or equivalent (e.g., GED)

o   Some college or no degree

o   Associate degree

o   Bachelor degree

o   Graduate degree

16. What is your race/ethnicity?

o   White

o   Black or African-American

o   Latino or Hispanic

o   American Indian or Alaskan native

o   Asian

o   Native Hawaiian or other Pacific Islander

o   From multiple races

o   Some other race (please specify) _______________________

17. Which of the following categories best describes your employment status?

o   Employed, working full-time

o   Employed, working part-time

o   Not employed, looking for work

o   Not employed, NOT looking for work

o   Retired

o   Disabled 

18. Do you have a car you can use?

o   Yes, I own a car

o   Yes, I lease a car

o   Yes, my family has a car

o   No, I must use public transportation

o   Prefer not to answer. 

19. What language do you mainly speak at home?

o   English

o   Spanish

o   Chinese

o   Russian

o   Vietnamese 

o   Some other language

20. If English is not the primary language spoken at home, is there someone who lives with you that reads, writes, and speaks English who could help you speak with a doctor over the phone or through your internet, phone, tablet, or computer. 

o   Yes

o   No 

o   N/A
